# Supplementary material for: Genome-wide identification and expression analysis of the ERF transcription factor family in pineapple (Ananas comosus (L.) Merr.)
Source: PeerJ. 2020 Sep 22;8:e10014. doi: 10.7717/peerj.10014 (PMC7518161; doi:10.7717/peerj.10014)
Supplement: Supplemental Information 1 — The location of the conserved YRG and RAYD elements are indicated by black bars. Black asterisks represent the 14th and 19th amino acid residues among the AP2/ERF domain proteins. [file peerj-08-10014-s001.pdf]

```
          1        10        20                  30        40        50
AcoERF1   CEYRGVRQRTWG.KWVAEIREPRK........RTR.IWLGSFATAEEAALAYDEAARRLYG.PDAFLNLP
AcoERF2   RKFRGVRRRPWG.KYAAEIRDPARG........VR.VWLGTFDTAEEAAMVYDSAALQLRG.PRAATNFS
AcoERF3   VRFRGVRRRPWG.RWAAEIRDPARR........RR.VWLGTFDTAEEAAAAYDSAAVRMRG.PRAVTNFP
AcoERF4   SEYLGVRRRPWG.RYAAEIRNPYTK........ER.HWLGTFDTAEEAAVAYDVTAISFSGPARARTNFY
AcoERF5   KLYRGVRQRHWG.KWVAEIRLPRN........RTR.LWLGTFDTAEDAALAYDRAAFRLRG.DSARLNFP
AcoERF6   ..YRGVRQRPWG.KWAAEIRDPRRA........VR.KWLGTFDTAEEAALAYDRAAIEFRG.PRAKLNFP
AcoERF7   CLYRGVRQRTWG.KWVAEIREPNR........GSR.LWLGTFPTAELAARAYDEAARAMYG.SLARVNFE
AcoERF8   TQYRGIRRRPWG.KWAAEIRDPGKG........VR.VWLGTFATPEDAARAYDRAARRIRG.KKAKLNFP
AcoERF9   GRFLGVRRRPWG.RYAAEIRDPTTK........ER.HWLGTFDTAQEAALAYDRAALSMKG.TQARTNFM
AcoERF10  GRFLGVRRRPWG.RYAAEIRDPTTK........ER.HWLGTFDTAHEAALAYDRAALSMKG.TQAKTNFM
AcoERF11  ..YRGVRMRSWG.SWVSEIRAPNQ........KTR.IWLGSYSTPEAAARAYDAALLCLKG.ASSSASLN
AcoERF12  .LYRGVRQRPWG.KWAAEIRDPRRA........AR.KWLGTFNTAEEAARAYDLAAIEFRG.ARAKLNFP
AcoERF13  KLYRGVRQRHWG.KWVAEIRLPKN........RTR.LWLGTFDTAEEAALAYDKAAFRLRG.DFARLNFP
AcoERF14  RRYRGVRQRPWG.KWAAEIRDPHKA........AR.VWLGTFDTAEGAARAYDEAALRFRG.SRAKLNFP
AcoERF15  TQYRGVRKRKWG.KWVSEIREPGK........KTR.IWLGSFESAEMAAAAHDVAALRLRG.RE..ARLN
AcoERF16  SRYRGIRQRPWG.KWAAEIRDPRKG........VR.VWLGTFNTPEEAARAYDVEARRIRG.KKAKVNFP
AcoERF17  VHFRGVRKRPWG.RFAAEIRDPWKK........TR.KWLGTFDTAEEAARAYDEAARNLRG.PKAKTNFG
AcoERF18  KRYVGVRQRPSG.RWVAEIKDTVQ........KIR.VWLGTFDTAEAAARAYDEAACLLRG.ANTRTNFW
AcoERF19  RHYRGVRRRPWG.KYAAEIRDSERHG.......AR.VWLGTFDTAEEAAAAYDRAAFRMRG.ARALLXWG
AcoERF20  SNYKGVRRRPWG.KFAAEIRDLSRRG.......AR.VWLGTFETAEEAALAFDVAALRMRG.PRTRLNFP
AcoERF21  KLYRGVRQRQWG.KWVAEIRLPQN........RVR.VWLGTYRPAKPNPKPNTNPNPSKKD.PPANDDSN
AcoERF22  PSYRGVRMRAWG.KWVSEIREPRK........KTR.IWLGTFPTPEMAARAHDAAALCVKG.PA..AALN
AcoERF23  RKFRGVRRRPWG.KYAAEIRDPVRG........VR.VWLGTFDTAEEAAMVYDSAALQLRG.PRAATNFS
AcoERF24  VRYRGVRKRPWG.RFAAEIRDPAKK........SR.VWLGTFDTAEDAARAYDAAALQFRG.PKAKTNFA
AcoERF25  SAYRGVRRRSSG.RWVAEIRDPIR........RVR.SWLGTYGSAEAAAEAYRAAAERFRA.EQKIG..G
AcoERF26  RPYIGVRKRPSG.KFAAEIRDSARGG.......ER.VWLGTFESAEAAALAYDQAAYAVRG.AAAVLNFP
AcoERF27  KLYRGVRQRQWG.KWVAEIRLPRS........RSRRLWLGTFDTAEDAALAYDREAFKLRG.ESARLNFP
AcoERF28  VRYRGVRQRPWG.RYAAEIRDPAKR........RR.VWLGTFDTPEAAARAYDAAALRFRG.PKAKTNFP
AcoERF29  PVYKGVRQRGGE.RWVCEVREPNNGPGGGARSNAR.IWLGTFPTAEMAARAHDVAALALRG.RA..ACLN
AcoERF30  VHYRGVRKRPWG.RYAAEIRDPWKK........TR.VWLGTFDTPEEAALAYDHAARSLRG.DKAKTNFP
AcoERF31  NQYRGIRQRPWG.KWAAEIRDPRKG........VR.VWLGTFNTAEEAARAYDVEARRIRG.KKAKVNFP
AcoERF32  PSYRGVRMRAWG.KWVSEIREPRK........KTR.IWLGTFPTPEMAARAHDAAALCVKG.PA..AALN
AcoERF33  GRYRGVRMRAWG.KWVSEIREPRK........KSR.IWLGTFPTPEMAARAHDAAALCVKG.AAARLRVK
AcoERF34  CPYRGVRQRTWG.KWVAEIREPNR........GAR.LWLGTFNTALEAAQAYDSAARNLYG.DCARLNLN
AcoERF35  RSYRGVRRRPWG.KFAAEIRDSTRHG.......VR.VWLGTFDSAEEAAMAYDQAAFAMRG.PAAVLNFP
AcoERF36  VHYRGVRKRPWG.RYAAEIRDPAKR........SR.VWLGTFDTAEDAARAYDAAALEFRG.PKAKTNFP
AcoERF37  RSYIGVRRRPWG.KFAAEIRDSTRNG.......AR.VWLGTFDTEEAAALAYDQAALSVRG.AAAVLNFS
AcoERF38  ....GARRRGR..VRGGDTRLEPP........WGP.HGLGTFDTAEEAARAYDRAAFAMRG.HLAVLNFP
AcoERF39  PRFRGVRRRPWG.KYAAEIRDPWRR........IR.VWLGTYNTAEEAAKVYDSAAIQLRG.PHATTNFS
AcoERF40  PKYKGVRQRKWG.RWAAEIRNPVS........GCR.EWLGTFDNAEAAAAAYQSAAKQFKA.KRSRN..A
AcoERF41  KHYRGVRQRPWG.KYAAEIRDPARNG.......AR.VWLGTFETAEEAALAYDRAAYRMRG.SRALLNFP
AcoERF42  NQYRGIRRRPWG.KWAAEIRDPRKG........VR.VWLGTYNTPEEAARAYDAEARRIRG.KKAKVNFP
AcoERF43  RLFRGVRMRKWG.TWVSEIRVPRS........QSR.IWLGSFDHPEKAARAYDAAVYCLRG.AA..GNFN
AcoERF44  KRFRGVRRRPWG.RWAAEIRDPTQR........KR.VWLGTFDTAEEAATVYDSAAIRLKG.PNAVTNFP
AcoERF45  PVYRGVRMRNWG.KWVSEIREPRK........KSR.IWLGTFPTAEMAARAHDVAALTIKG.QS..AHLN
AcoERF46  RRYRGVRQRPWG.KFAAEIRDPNRRG.......SR.VWLGTFDTAVEAARAYDRAAFKMRG.CKAILNFP
AcoERF47  KHYRGVRQRPWG.KFAAEIRDPAKNG.......AR.VWLGTFETAEDAALAYDRAAYRMRG.SRALLNFP
AcoERF48  KKFIGVRQRPSG.RWVAEIKDSSQ........KVR.LWLGTFDTPEDAARAYDDAARSLRG.ANARTNFG
AcoERF49  PVYKGVRRRGAD.RWVCEVREPNK........KSR.IWVGTFPTPEMAARAHDAAALALRG.AS..APLN
AcoERF50  RRFVGVRQRPSG.RWVAEIKDSAQ........RVR.LWLGTFDTAEAAARAYDDAARTLRG.TNARTNFN
AcoERF51  KKFRGVRQRHWG.SWVSEIRHPLL........KRR.VWLGTFETAEEAARAYDEAAVLMSG.RNAKTNFP
AcoERF52  PAYRGVRMRNWG.KWVSEIREPRK........KSR.IWLGTFPTAEMAARAHDAAARAVKG.AA..AVLN
AcoERF53  MRYLGVRRRPWG.RYAAEIRDPATK........ER.HWLGTFDTAEEAAVAYDRAARSLRG.PRARTNFA
AcoERF54  KHYRGVRRRPWG.KWAAEIRDPNKA........AR.VWLGTFDTAERAAMAYDEAALRFKG.SKAKVNFP
AcoERF55  KLYRGVRQRHWG.KWVAEIRLPRN........RTR.LWLGTFDTAEDAALAYDREAFKLRG.ENARLNFP
AcoERF56  PVYRGVRSRNGG.RWVCEVREPHK........KSR.IWLGTYPTPEMAARAHDVAALALRG.EA..APLN
AcoERF57  RGYRGVRKRAWG.RWSAEIRDRVGR........CR.HWLGTYDTAEEAARAYDAAARGMRG.SKARTNFA
AcoERF58  TKFVGVRQRPSG.RWVAEIKGTTQ........KIR.MWLGTFETAEDAARAYDEAARIMCG.PRARTNFA
AcoERF59  ARYLGVRRRPWG.RYAAEIRDPATK........DR.HWLGTYDTAEEAAVAYDRAARALRG.PRARTNFA
AcoERF60  RHYRGVRQRPWG.KWAAEIRDPKKA........AR.VWLGTFDTAEEAAIAYDEAALRFKG.TKAKLNFP
AcoERF61  ERYRGVRRRPWG.RFAAEIRDPRRRGG.....GSR.VWLGTFDSAVAAARAYDRAAFAMRG.AKAILNFP
AcoERF62  KKYKGVRRRRWG.KWVSEIRVPGT........RHR.LWLGSYATAEAAAVAHDAAVFFLRG.PDAAEGFN
AcoERF63  RRYRGVRQRPWG.KWAAEIRDPHKA........AR.VWLGTFDTAEAAARAYDAAALRFRG.SRAKLNFP
AcoERF64  RRYKGVRRRKWG.KFVSEIRLPNS........RNR.IWLGSYDTPEKAARAFDAAAVCLRG.PLGSGRLN
AcoERF65  NQYRGIRQRPWG.KWAAEIRDPRKG........VR.VWLGTFNTAEEAARAYDAEARRIRG.KKAKVNFP
AcoERF66  TKFVGVRQRPSG.RWVAEIKGTTQ........KIR.MWLGTFETAEEAARAYDAAACLLRG.ANTRTNFL
AcoERF67  ARFKGVRRRKWG.KWAAEIRNPKT........RTR.LWLGTFDTAEAAAAAYQAASRRFAE.EL.....A
AcoERF68  PVYKGVRRRGAAGRWVCEVREPNK........KSR.IWLGTFPTAEMAARAHDVAAIALRG.RS..ACLN
AcoERF69  ................MGLRNSAS........SI.........TAEDAARAYDEAARLMCG.PRARTNFP
AcoERF70  SKFVGVRQRPSG.RWVAEIKDTTQ........KIR.MWLGTFETAEEAARAYDEAACLLRG.SNTRTNFV
AcoERF71  SGYRG.VQRSWR.KWVAEIRERRA........HAK..WLGTFATAEDAARAYDRAALVLYG.PRAHLNLQ
AcoERF72  TKYRGVRRRPWG.RYAAEIRDPQSK........ER.RWLGTFDTAEQAACAYDIAARAMRG.LKARTNFP
AcoERF73  TKYRGVRRRPWG.RYAAEIRDPQSK........ER.RWLGTFDTAEQAACAYDIAARAMRG.LKARTNFP
AcoERF74  KHYRGVRRRPWG.KWAAEIRDPNKA........AR.VWLGTFDTAERAAMAYDEAALRFKG.SKAKVNFP
                         *      *
```

YRG element

RAYD element
