# Supplementary figures and images for: Genome-wide identification and expression analysis of the ERF transcription factor family in pineapple (Ananas comosus (L.) Merr.)
Source: PeerJ. 2020 Sep 22;8:e10014. doi: 10.7717/peerj.10014 (PMC7518161; doi:10.7717/peerj.10014)

Motif 1

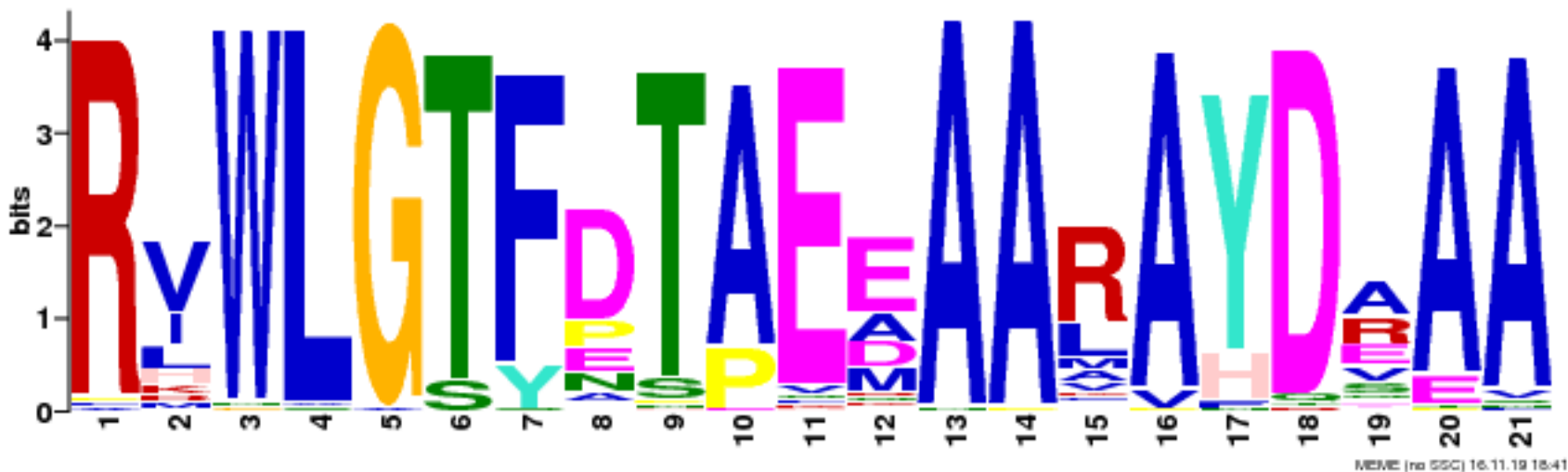

Motif 2

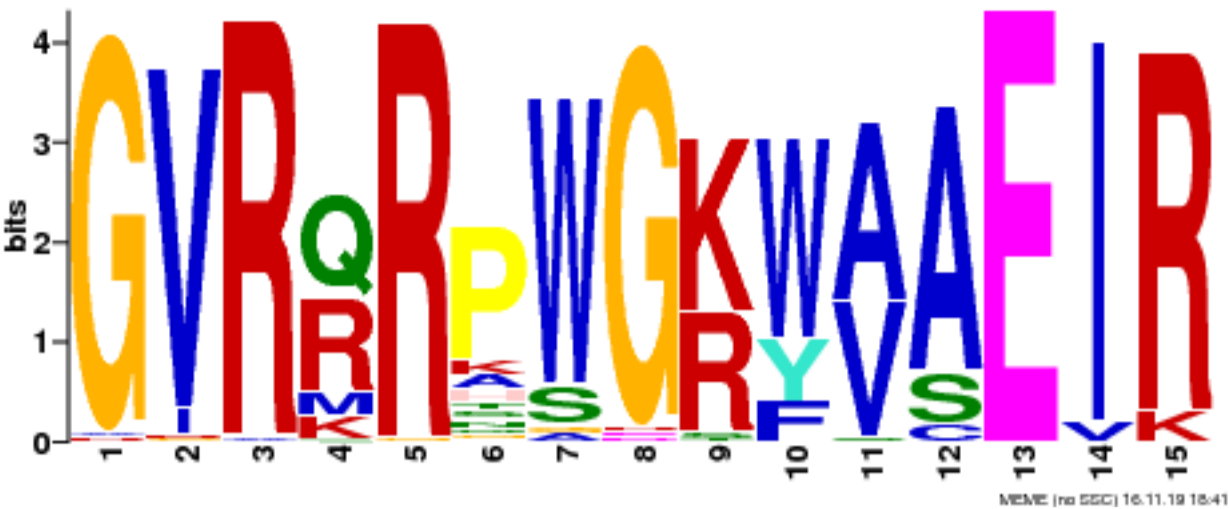

Motif 3

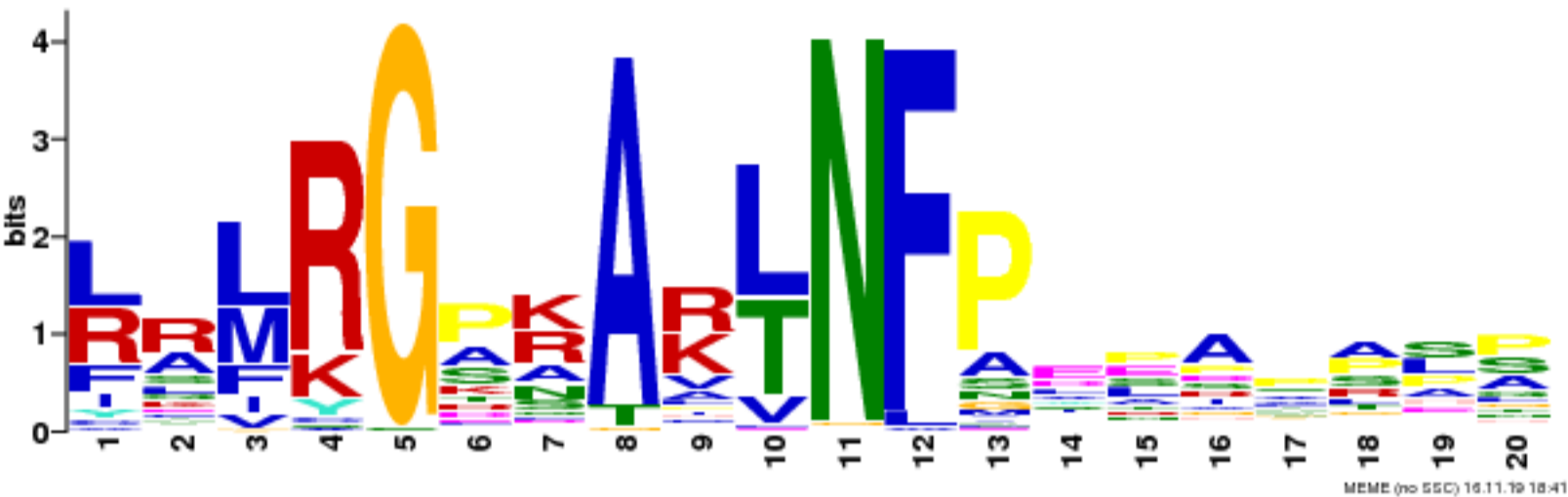

Motif 4

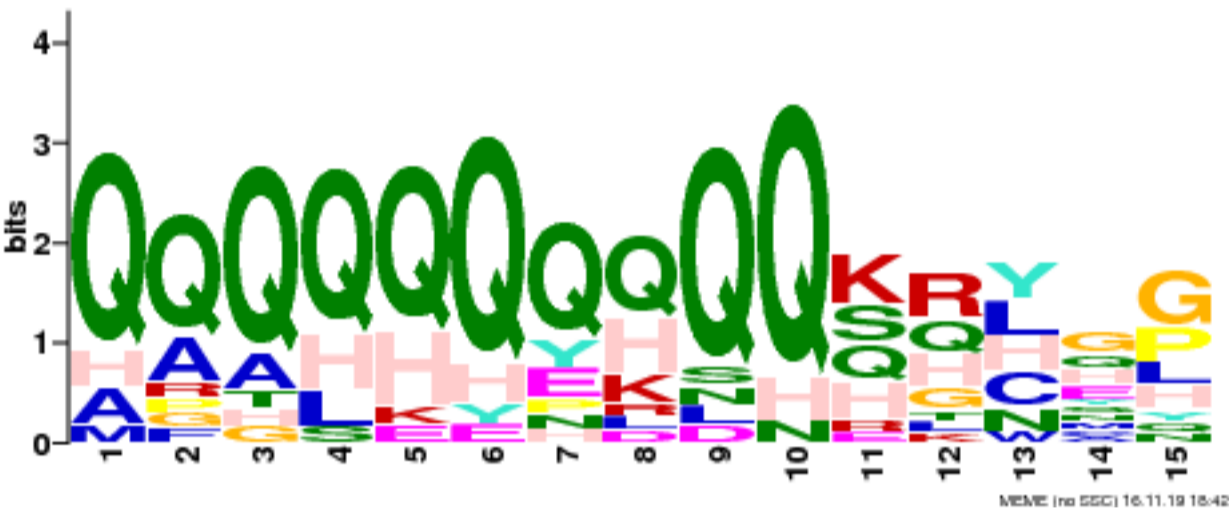

Motif 5

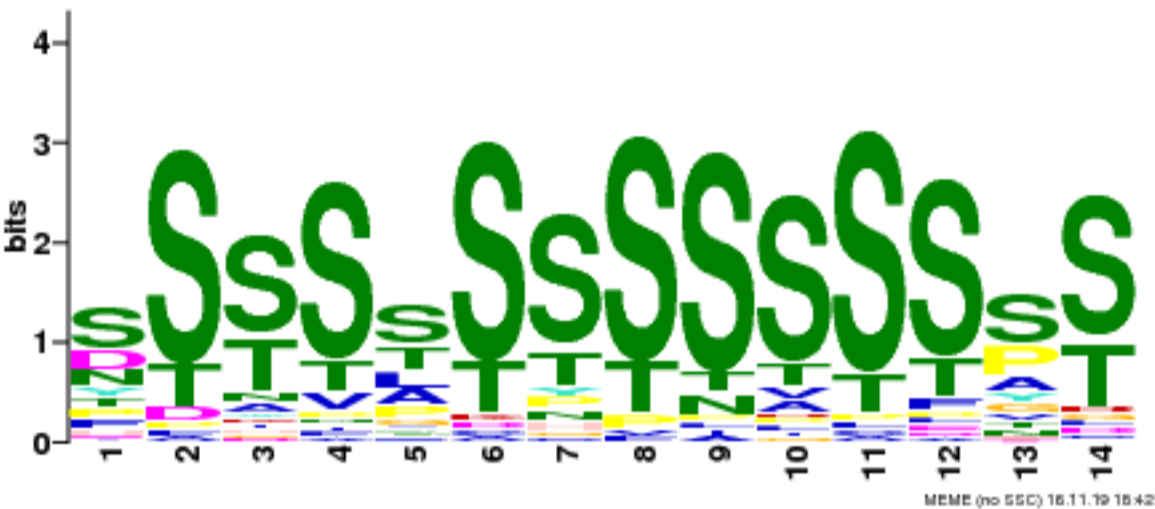

Motif 6

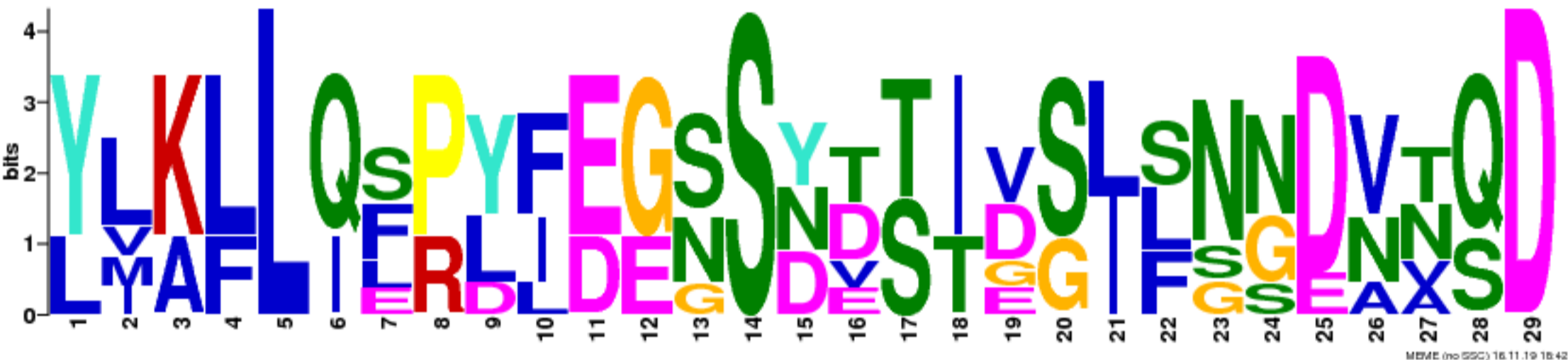

Motif 7

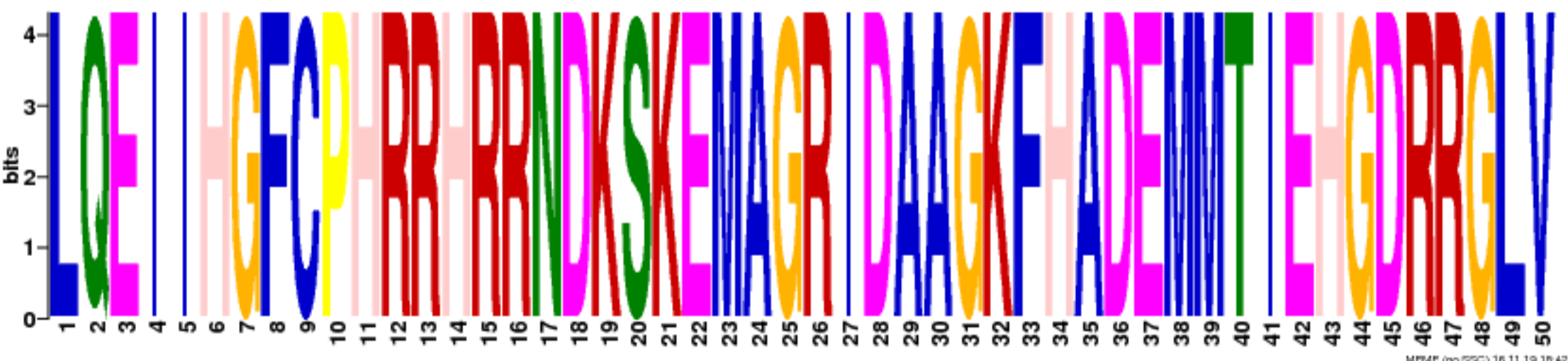

Motif 8

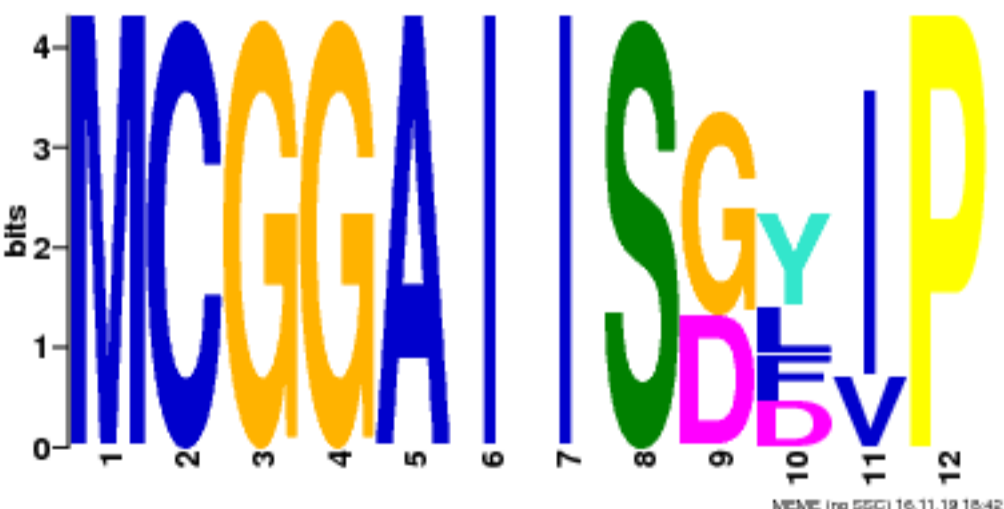

Motif 9

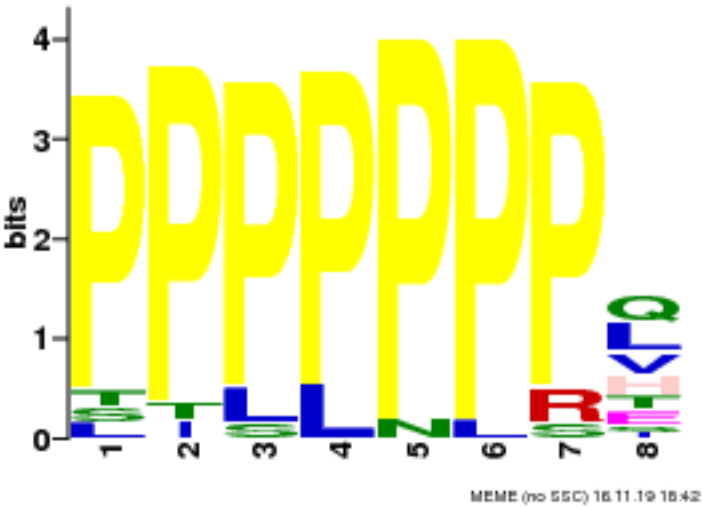

Motif 10

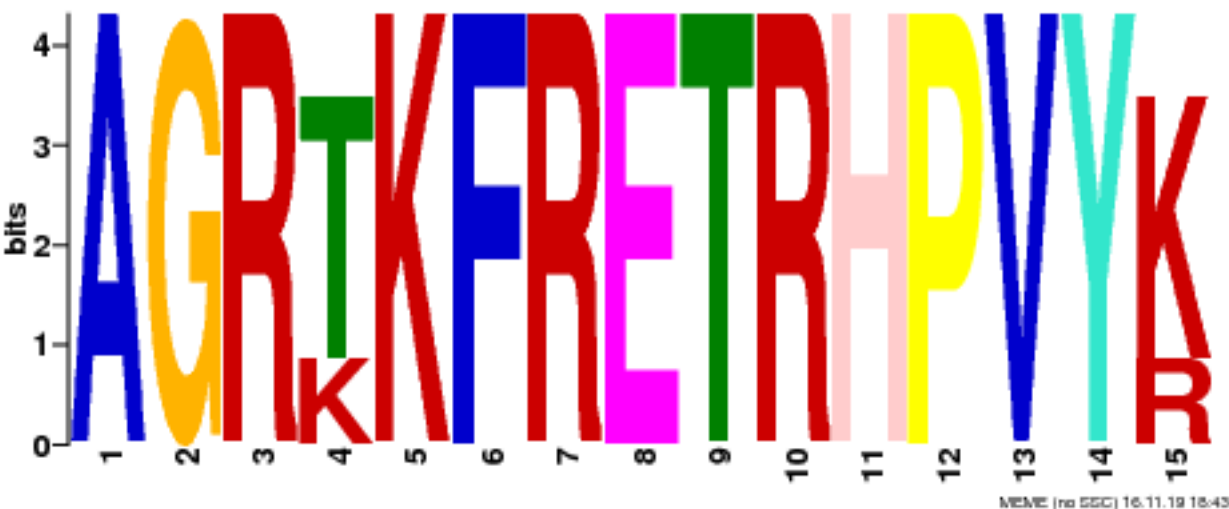

Supplement: Supplemental Information 2 [file peerj-08-10014-s002.pdf]

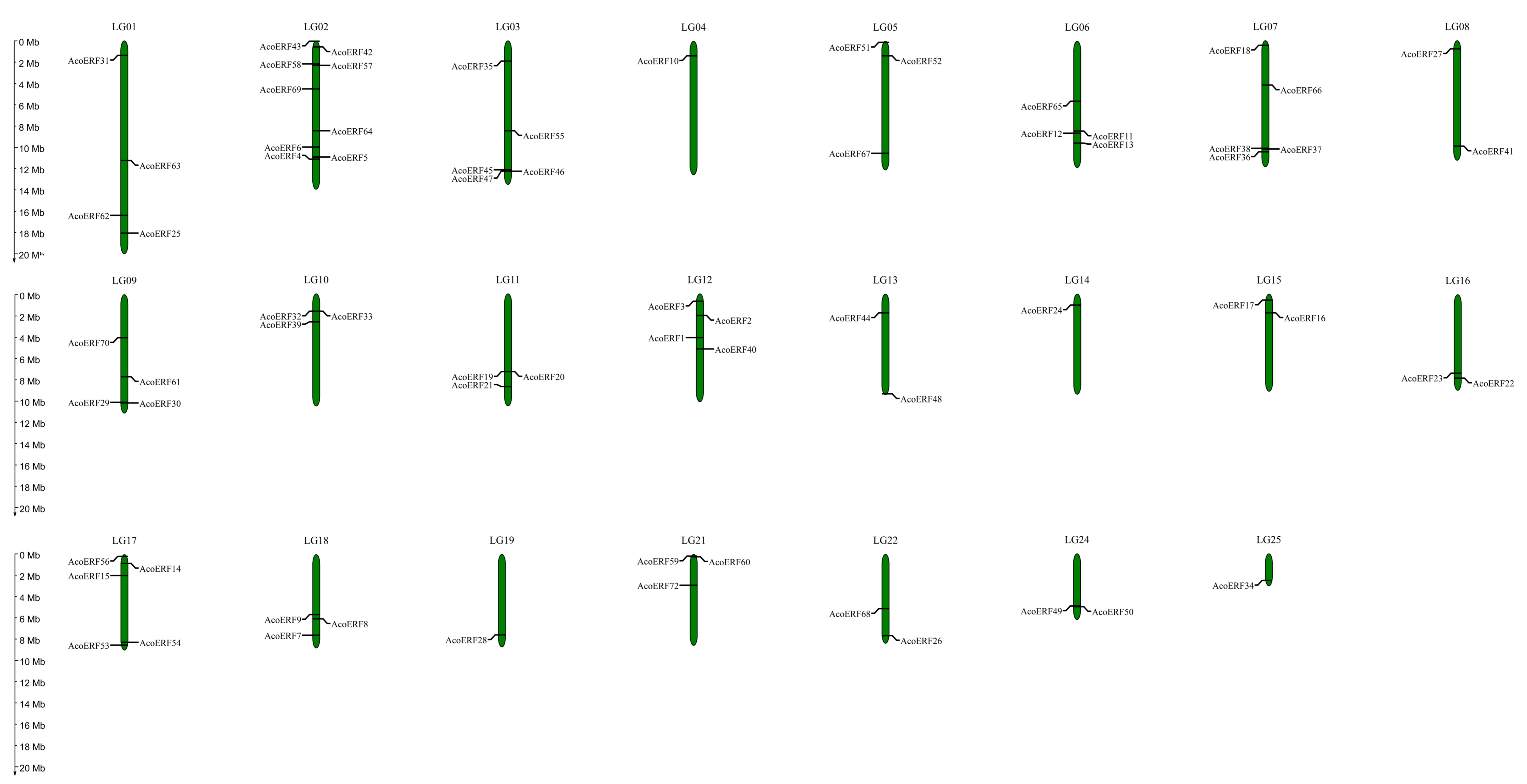

Supplement: Supplemental Information 3 — The chromosome number is indicated above each bar and the length of the bar represents the size of the chromosome in pineapple. Gene star position is shown on chromosome. [file peerj-08-10014-s003.pdf]
